# Measurement of individual differences in face-identity processing abilities in older adults

**DOI:** 10.1186/s41235-021-00310-4

**Published:** 2021-07-18

**Authors:** Isabelle Boutet, Bozana Meinhardt-Injac

**Affiliations:** 1 School of Psychology, University Ottawa, Ottawa, Canada; 2 Department of Psychology, Catholic University of Applied Sciences Berlin (KHSB), Köpenicker Allee 39–57, 10318 Berlin, Germany

**Keywords:** Aging, Face memory, Face matching, Neuropsychological assessment, Social cognition

## Abstract

**Background:**

Face-identity processing declines with age. Few studies have examined whether face-identity processing abilities can be measured independently from general cognitive abilities in older adults (OA). This question has practical implications for the assessment of face-identity processing abilities in OA and theoretical implications for the notion of face processing as a specific ability. The present study examined the specificity of face memory and face matching abilities in OA aged 50 + .

**Methods:**

Performance of younger adults (YA) and OA was measured on face tasks: Cambridge Face Memory Task (CFMT), the Glasgow Face Matching Task (GFMT), holistic processing; and tasks of general cognition: fluid intelligence, selective attention, and mental rotation. Data were analyzed using multiple regression models encompassing (i) the CFMT/GFMT and measures of general cognition; and (ii) all face processing tasks.

**Results:**

Across the two age groups, models encompassing all face tasks were significant and accounted for more variance in the data than models encompassing the CFMT/GFMT and measures of general cognition. General cognitive abilities accounted for 17% of variance for the GFMT (*p* < 0.01) and 3% for the CFMT (*p* > 0.05).

**Discussion:**

Our results suggest that face memory can be measured independently from general cognition using the CFMT in OA. Implications for the notion of a general face processing factor across the adult lifespan are discussed.

**Supplementary Information:**

The online version contains supplementary material available at 10.1186/s41235-021-00310-4.

## Significance statement

Face-identity processing abilities decline with age (e.g., reviewed by Boutet et al., 2015; Germine et al., 2011; Hildebrandt et al., 2011). These age-related impairments have consequences for older adults’ ability to engage in social interactions and for real-life contexts such as the reliability of older eye-witnesses to crime (Searcy et al., 2000) and professions that require face identification (e.g., passport officers; Wirth & Carbon, 2017). We examined the extent to which face matching and face memory can be measured independently of general cognitive functions in older adults (OA). Performance of younger adults (YA) and OA was measured on several tasks of face processing, including the Cambridge Face Memory Task (CFMT) and the Glasgow Face Matching Task (GFMT), as well as measures of general cognition such as fluid intelligence and attention. Models testing associations among face tasks and associations between face tasks and general cognitive abilities were tested. If age-related decline in other cognitive abilities contaminates scores on tests of facial identity processing, then poor performance could be interpreted (erroneously!) as evidence for impaired face processing. Our results suggest that face memory can be measured independently from general cognition using the CFMT in OA. Our results also extend findings in YA by supporting the existence of a specific face processing factor across the adult lifespan (McCaffery et al., 2018; Verhallen et al., 2017; Wilmer, 2017).

## Introduction

Face-identity processing abilities start to decline as early as 30 years of age, and robust age-related differences have been reported for a variety of facial identity processing tasks in older adults (OA) aged 65 + (e.g., reviewed by Boutet et al., 2015; Germine et al., 2011; Hildebrandt et al., 2011; Susilo et al., 2013). Impairments in face discrimination and face recognition can have negative consequences for social interactions and real-life contexts. Uncertainty regarding an individual’s identity, together with the embarrassment associated with errors in person recognition, can impede social engagement and have negative consequences for physical and psychological well-being (e.g., Avery et al., 2016). Moreover, deficits in face processing can reduce the reliability of eye witness testimony and hinder face identification performance in professional contexts (e.g., police and passport officers; Wirth & Carbon, 2017). Finally, disturbances in social cognitive abilities, including face recognition, can signal the onset of neurodegenerative disease (Henry et al., 2016).

Most studies that have examined the impact of aging on face-identity processing have focused on group-mean differences and have utilized lab-based measures. This contrasts with research with younger adults (YA) where there is a growing interest in developing psychometrically sound tests (e.g., Cambridge Face Memory Test, Duchaine & Nakayama, 2006) that can be utilized to measure variations in face-identity processing and advance our understanding of this important social cognitive ability (Wilmer, 2017). In contrast, few studies have measured determinants of individual differences in facial identity processing in OA (Hildebrandt et al., 2011; Schretlen et al., 2001). The goal of the present study was to address this gap by examining the extent to which face matching and face memory can be measured independently of general cognitive functions in OA.

Research conducted with YA suggests that previously popular measures such as the Benton Face Recognition Test (Benton & Van Allen, 1968) and the Warrington Recognition Memory Test for Faces (Warrington, 1984) have limited utility for clinical and research contexts (Duchaine & Weidenfeld, 2003; Duchaine & Nakayama, 2004, 2006, but see Rossion & Michel, 2018; Mishra et al., 2020, for evidence supporting the validity of some versions of the BFRT). This prompted the development of new tests, such as the Cambridge Face Memory Test (CFMT; Duchaine & Nakayama, 2006) and the Glasgow Face Matching Test (GFMT; Burton et al., 2010). Performance on the CFMT (Beaudoin & Desrichard, 2011) and GFMT (Verhallen et al., 2017 but see McCaffery et al., 2018) correlates with self-report evaluations of face processing abilities, suggesting that these tests have good construct validity. Moreover, internal reliability is considered good for both tests (Verhallen et al., 2017).

Studies conducted using the CFMT and/or GFMT have revealed significant associations between different measures of face-identity processing (McCaffery et al., 2018; Verhallen et al., 2017; Wilmer, 2017; see also Bowles et al., 2009; Gignac et al., 2016). Moreover, these associations tended to be larger than associations between identity-processing and other cognitive abilities. These findings have been interpreted as evidence for a general face processing factor (*f*) which is akin to *g*, the common factor that is thought to underlies scores on different sub-tests of intelligence (Verhallen et al., 2017). Such specificity is congruent with evidence that specialized cognitive and biological mechanisms are responsible for processing human faces (e.g., Gauthier, 2018; Haxby et al., 2000).^1^

Only two of the aforementioned studies included individuals aged 65 + in their sample and the number of OA tested was small (Bowles et al., 2009; McCaffery et al, 2018 Study 2). Studies focusing on OA utilized older tests whose properties have since been questioned (e.g., Benton Face Recognition Test, Schretlen et al., 2001) or lab-based tests (Hildebrandt et al., 2011). Given that age is a key determinant of individual differences in face-identity processing, paucity of research with OA limits our understanding of this critical ability. At a theoretical level, paucity of research with OA limits the generalizability of claims made about the specificity of face processing across the adult lifespan (McCaffery et al., 2018; Verhallen et al., 2017; Wilmer, 2017). At a practical level, it limits interpretation of scores of OA on tasks of face-identity processing. Indeed, being able to isolate decline in a specific cognitive ability from decline in other related abilities is an important challenge in measurement of geriatric populations (e.g., Miyake et al., 2000). This issue of *task impurity* has received limited attention in the literature on face-identity processing and aging (Gignac et al., 2016; Hildebrandt et al., 2011; Schretlen et al., 2001). Yet, if age-related decline in other cognitive abilities contaminates scores on tests of facial identity processing, then poor performance could be interpreted (erroneously!) as evidence for impaired face processing.

Measurement of face-identity processing may be less specific in older adults for several interrelated reasons. First, correlations between and among distinct cognitive abilities can increase with age (e.g., Li et al., 2004; but see Tucker-Drob & Salthouse, 2008). Second, additional brain regions can be recruited when OA perform the same tasks as YA. In the case of faces, these changes appear to arise from de-differentiation of the ventral network specialized for identity processing (e.g., Zebrowitz et al., 2016) and/or the recruitment of compensatory prefrontal mechanisms (e.g., Burianová et al., 2013). Finally, some studies with YA have reported small correlations between the CFMT or GFMT and general cognitive abilities such as executive functions (McCaffery et al., 2018). Given that these abilities are known to decline with age (reviewed by Reuter-Lorenz et al., 2016), one might expect relationships between these measures and general cognitive abilities to increase with age.

### The present study

We investigated associations between different measures of face-identity processing and between measures of face-identity processing and measures of general cognitive function in YA and OA. Face tasks included the CFMT, the GFMT, and a measure of holistic face processing (composite task). Holistic processing (HP) is defined as automatic processing of individual face parts into a whole or Gestalt. Many studies on individual differences in YA have included a measure of HP in their design because it is considered a hallmark of what makes faces ‘special’ (reviewed by Gauthier, 2020).

Three measures of general cognitive function were included. Fluid intelligence and selective attention were measured because these abilities are core aspects of executive functions. These two abilities are known to decline with age and hence are likely to contribute to individual differences in OA (fluid intelligence, e.g., Finkel et al., 2003; selective attention, e.g., Greenwood et al., 1993). Mental rotation was also measured because this ability declines with age (e.g., Techentin et al., 2014), is linked with impaired face recognition in individuals with Alzheimer’s disease (Adduri & Marotta, 2009), and is related to fluid intelligence (Tachibana et al., 2014; Varriale et al., 2018). Finally, visual acuity was measured in our OA sample because age-related decrements in basic visual abilities (e.g., Sekuler & Picciano Hutman, 1980) have been linked to performance on face tasks (Boutet et al., 2020; Davidson et al., 2019; Owsley et al., 1981).

Data were analyzed using multiple regression models. A series of models focused on whether general cognitive abilities are significant predictors of performance on the CFMT/GFMT. Finding that tests of general cognitive abilities are significant predictors of performance on the CFMT or GFMT would suggest that this test does not measure face-identity processing independent from general cognitive abilities. Another series of models focused on associations between different measures of face processing, which would be consistent with the existence of a general face processing factor (McCaffery et al., 2018; Verhallen et al., 2017).

Finally, we report indexes of internal reliability for the CFMT and GFMT in our sample. There is a paucity of information on the internal reliability of the CFMT, GFMT and composite task in OA. Yet, reporting reliability statistics is important in individual differences studies because reliability sets limits on how strongly a measure can correlate with other measures (Wilmer, 2008). Furthermore, one cannot assume that reliability indexes reported for YA generalize to OA since the two groups may rely on different strategies with varying stability to perform these tasks.

## Materials and methods

### Participants

142 YA (range: 20–30 years of age; mean: 23.1, SD = 2.5; 105 female) and 118 OA (range: 51–85 years of age; mean: 66.1, SD = 8.1; 63 female) were recruited. Data from 2 YA and 16 OA were excluded because of missing values. None of the participants reported having any medical condition or taking any medication that would interfere with the experiment. Participants received a small monetary compensation for travelling costs (10 €) or course credit for participation. According to the Declaration of Helsinki, written informed consent was obtained from all participants. The experimental procedures for the project were approved by the local ethics committee of Mainz University (protocol number: 2017-JGU-psychEK-009).

### Materials

#### Cambridge face memory test (CFMT)

**Fig. 1:**
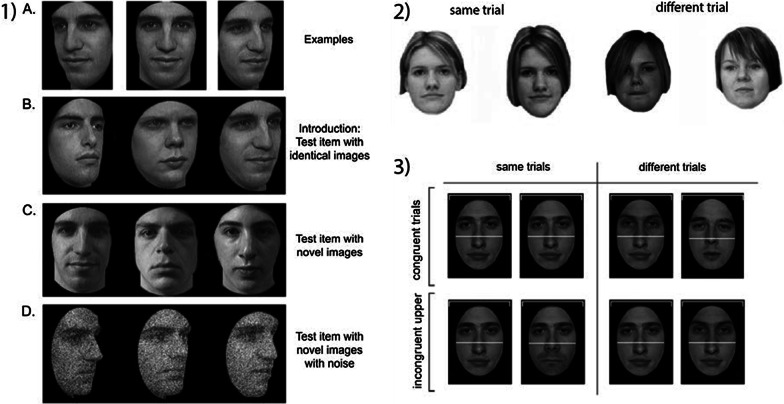
(1) The Cambridge Face Memory Task (CFMT; Reproduced with permission from Duchaine & Nakayama, 2,006). **A** At learning, six target faces are shown for memorization followed by test trials where the target face has to be identified among two distractors. Recognition of target faces is tested in three stages with increasing difficulty. **B** Learned faces are shown in the same viewpoint as learning. **C** Learned faces are shown in a different viewpoint than at learning. **D** Learned faces shown in different viewpoints and with noise added to the images. (2) The Glasgow Face Matching Task (GFMT; The individuals shown in the figure have given written informed consent to publish these images). Participants are asked to determine the two simultaneously presented faces are matching (yes/no). Presentation time is self-paced. (3) The complete composite test. Examples of same and different congruent and incongruent trials. Participants indicate whether the identity of the relevant half (top or bottom) is same or different. In the examples, the relevant half is always the top, as indicated by the white horizontal cue. In congruent same trials, the identity of the relevant and irrelevant halves of two sequentially presented faces is the same. In congruent different trials, the identity of the relevant and irrelevant halves of two sequentially presented faces is different. In incongruent same trials, the identity of the relevant halves of two sequentially presented faces is the same, but the identity of the two irrelevant face halves is different. In incongruent different trials, the identity of the relevant halves of two sequentially presented faces is different, but the identity of the two irrelevant face halves is the same. Because of automatic holistic processing of faces, the presence of irrelevant face features interferes with matching of the relevant halves, leading to poorer performance in incongruent than congruent trials

The CFMT measures short-term memory for unfamiliar faces (see Duchaine & Nakayama, 2006, for details; Fig. 1). The test consists of three stages: introduction/same images, novel images and novel images with noise. For the introduction/same images stage, three study images are shown for 3 s each in the views illustrated in Fig. 1. Three test items are then presented and participants are instructed to pick out the individual whom they were just shown. Participants select the target via key press. Each test item includes an item identical to a study item. The same procedure is repeated for a total of 6 target faces. For the novel images stage, participants are presented with a single front view of each target face. They are given 20 s to review this image. Following the review image, participants are presented with 30 forced-choice test items (6 target faces × 5 presentations) in random order. Each test consists of three faces, one of which is a target. All are novel images in which the lighting, pose, or both vary. For the novel images with noise stage, participants are presented with the review image again for 20 s. Following this, 24 test items (6 target faces × 4 presentations) are presented in a random order. These items consist of novel images with different levels of nose added to the face images. Internal consistency reliability of the CFMT was good in both age groups (Cronbach’s *α* = 0.86 in YA and *α* = 0.85 in OA). Face stimuli measured 4 × 6 cm (average across targets and viewpoints) on screen.

#### Glasgow face matching test (GFMT)

The GFMT measures unfamiliar face matching. The short version of the GFMT was administered (see Burton et al., 2010, for details; Fig. 1). In ‘match’ trials, two images of the same individual are taken from different camera angles, which limits discrimination based on image-matching strategies (Bruce, 1982; Hancock et al., 2000). 20 different and 20 same trials were tested in random order. Each face measured 12 × 14 cm on screen. Split-half reliability of this task has been reported at *r* = 0.91 (Burton et al., 2010). In our study, internal consistency reliability of the GFMT was acceptable in YA (Cronbach’s α = 0.71 and questionable in OA (Cronbach’s α = 0.63).

#### Holistic processing (HP)

An adaptation of the complete composite test was used (Wang et al., 2016; see Meinhardt et al., 2014 for details; see Fig. 1). Composite faces are constructed by putting together the top half of one face with the bottom half of another face. Participants determine whether the upper or the lower face halves in two successively presented composite images are the same or different. A cue is presented to indicate which half was relevant for a given trial. The trial sequence was as follows: a fixation point was shown for 750 ms, followed by a blank screen for 300 ms, first stimulus was shown for 800 ms, followed by a mask for 400 ms, and a blank screen for 800 ms, second stimulus was shown for 386 ms, followed by a mask for 400 ms, a blank screen remained until a response was provided. Stimuli measured 9 × 12 cm on screen. 32 congruent and 32 incongruent trials (half same, half different) were shown in random order. HP was operationalized as residuals between congruent and incongruent conditions in the composite task because this index tends to be more reliable (DeGutis et al., 2013). Reliability of this task was good in YA (Cronbach’s *α* = 0.82) and acceptable in OA (Cronbach’s *α* = 0.71).

#### Selective attention (SA)

Selective attention was measured using a superposition paradigm (see Meinhardt et al., 2019 for more details). Participants are shown faces and houses that are overlaid using a transparency. Participants are asked to categorize the gender of the face, which requires selective attention to the face and attentional suppression of the irrelevant house. The irrelevant, to be ignored house, was overlaid on the face stimuli using two levels of opacity: 35% (low opacity, LO) and 65% (high opacity, HO). In the LO condition, the distractor houses have low salience and therefore produce low levels of competition, while in the HO condition, the distractor houses are much more salient and produce higher levels of attentional competition. Stimuli measured approximately 10 × 14 cm on screen. We chose this task because it uses faces as stimuli, which offers a better comparison with the other face tasks given that faces attract attentional and motivational resources (e.g., Langton et al., 2008). Participants are tested at two levels of difficulty (low and high opacity) with 30 trials per level (total of 60 trials). Stimulus presentation was self-paced. Reliability was questionable in YA (Cronbach’s *α* = 0.27) and OA (Cronbach’s *α* = 0.32). Selective attention was operationalized as residuals between low vs. high opacity conditions.

#### Mental rotation (MR)

We used a subtest from the basic module of the IST-2000-R (see Liepmann et al., 2007 for more details). The test items were identical to the original test but presented on a computer. Cronbach’s α typically range from 0.88 to 0.98 suggesting excellent test reliability (Liepmann et al., 2007). For each trial, a target cube is shown along with five test cubes presented in a row below the target. The cubes have different surfaces (e.g., points, lines, etc.). Only one of the eight test cubes corresponds to the target cube, but in rotated form. The participants are asked to select the test cube which matches the target cube. Participants had 10 min to complete up to 12 trials.

#### Fluid intelligence (FI)

A short version of Raven’s standard progressive matrices task (Raven, 2000) was used to measure abstract nonverbal reasoning, which is a key component of fluid intelligence. Cronbach’s α alpha for the short version is 0.65 (Arthur & Day, 1994). In this test, all trials have a visual-geometric design with a missing piece. Participants choose one out of eight elements to complete the matrix. Stimulus presentation lasts until a response is provided. Participants had 10 min to complete up to 40 matrices.

#### Acuity

High contrast visual acuity was measured using the Freiburg Visual Acuity Test (FrACT) (Bach, 1996). The FrACT uses an adaptive method (Best PEST) to assess a visual threshold, producing acuity ratios ranging from 0.05 (lowest possible score, 20/400 ft. ≈ 6/120 m) to 2.0 (highest possible score, 20/10 ft.,  ≈ 6/3 m). Participants completed this test from a viewing distance of 300 cm.

#### Apparatus

All tasks were run using Inquisit software. Stimuli were displayed on NEC Spectra View 2090 TFT display with 1600 × 1200 resolution and a refresh rate of 60 Hz. Stimuli were viewed binocularly at a distance of 70 cm. Participants were positioned using a distance marker, but no chin rest was used. Participants responded by pressing a button on an external key-pad.

### Procedure

All tests were administered via computer at the University of Mainz. Test order was counterbalanced across participants.^2^ Each test took 5–10 min to administer. Participants could take breaks as needed. All responses were recorded via left–right mouse presses or on predefined on-screen arrays. Prior to testing, participants read the testing instructions on the computer screen and asked questions if needed. For each test, practice trials were provided to familiarize participants with task requirements and response procedures. Up to three participants were tested at a time in the same experimental room, separated by movable partition walls. Inquisit 4.0 (Millisecond Software, Seattle, Washington) was used for programming computer-based test administration.

## Results

The estimated sample size required for a power of 0.90 for multiple regression models was calculated with Statistica 12.0 (TIBCO Software Inc, Palo Alto, California) using following parameters: explained variance *P*^2^ = 0.25, null hypothesized value of *C*^2^ = 0.05, *α* = 0.05 and 3 predictors. The estimated required sample was 118 participants. Our sample size is therefore adequate for interpretation of the regression models.

### Descriptive statistics & age-related effects

**Fig. 2:**
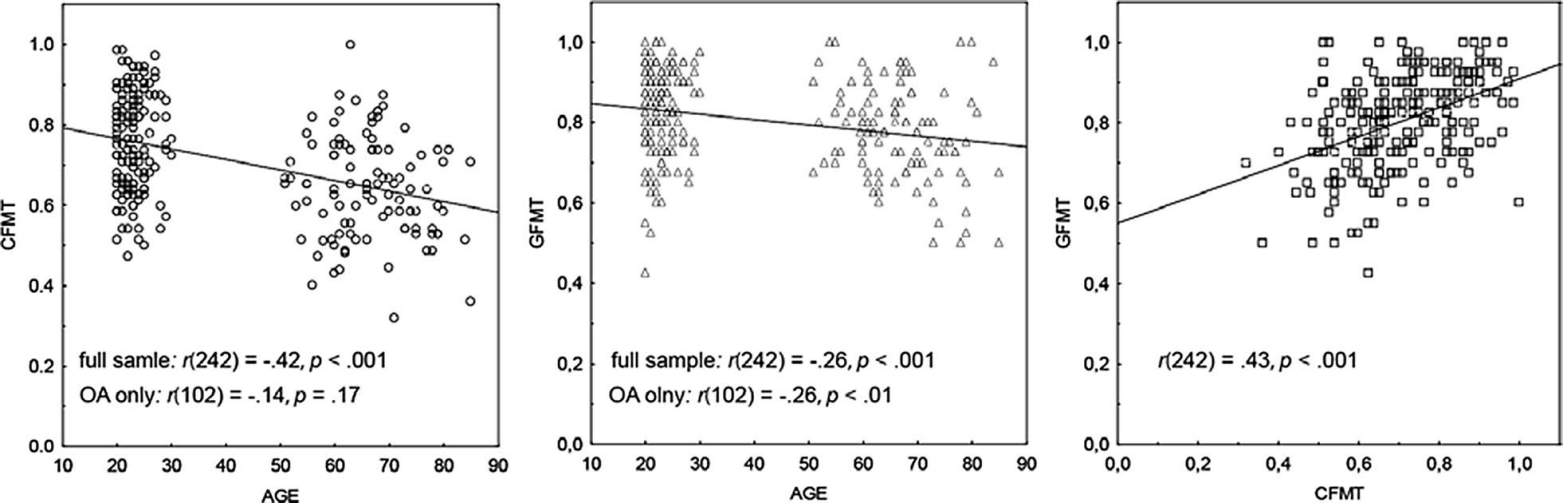
Scatterplots showing relationships between Age and the Cambridge Face Memory Test (CFMT) (left), between Age and the Glasgow Face Matching Test (GFMT) (middle) and between the CFMT and GFMT (right). *r* = Pearson’s correlation coefficient for the full sample and for older adults (OA) only. *r* is not reported for YA because of the very narrow age-range for this group. Spearman’s rank correlations are provided in Additional file 1: Tables 1, 2

Descriptive statistics and results of independent groups *t*-tests comparing YA to OA are provided in Table 1.^3^ OA performed more poorly than YA on all tasks except for the composite task, where significant age differences arose from larger holistic effects (calculated as residuals) in OA as compared to YA. Scatter plots illustrating correlations between the two face perception tasks and age are showing in Fig. 2.

**Table 1:** Mean Performance (M) and Standard Deviation (SD) in Young and Older Adults Groups, as well as age-related differences, for all tasks used in this study

	YA				OA				Mean across both groups				*t-*Test	
	*M*	*SD*	*Min*	*Max*	*M*	*SD*	*Min*	*Max*	*M*	*SD*	*Min*	*Max*	*t(240)*	*p*
CFMT	.76	.12	.47	.98	.64	.13	.32	1.0	.71	.13	.32	1.0	7.1	< .001
GFMT	.82	.10	.42	1.0	.77	.11	.50	1.0	.80	.11	.42	1.0	3.7	< .001
HP_CC	.89	.07	.56	1.0	.79	.11	.42	.97	.85	.10	.42	1.0	–	–
HP_IC	.72	.10	.40	.92	.60	.12	.30	.95	.67	.12	.32	.95	–	–
HP-res	.03	.07	− .26	.20	− .03	.11	− .37	.17	.0	.09	− .36	.20	5.1	< .001
HP-diff	.16	.11	− .03	.57	.18	.15	− .17	.57	.17	.13	− .17	.57	− 1.14	–
SA_LO	.94	.08	.06	1.0	.90	.14	.03	1.0	.93	.11	.03	1.0	–	–
SA_HI	.89	.09	.09	1.0	.76	.13	0.0	1.0	.83	.13	0.0	1.0	–	–
SA_res	− .01	− .06	− .37	.11	.03	.10	− .49	.24	.001	.08	− .49	.24	− 4.9	< .001
MR	.75	.23	.08	1.0	.61	.19	.08	1.0	.69	.22	.08	1.0	4.7	< .001
FI	.75	.11	.41	1.0	.43	.15	.05	.83	.62	.20	.05	1.0	17.9	< .001
Acuity	–	–	–	–	.95*	.36	.56	2					–	–

*YA* Younger Adults, *OA* Older adults, *GFMT* Glasgow Face Memory Test, *CFTM* Cambridge Face Matching Test, *HP_CC/HP_IC* Holistic Processing congruent/incongruent condition (raw data), *HP-diff* Holistic Processing (difference measure), *HP_res* Holistic processing (residuals), *SA-LO/SA-HI* Selective Attention low/ high opacity (raw data), *SA_res* Selective Attention (residuals), *MR* Mental Rotation, *FI* Fluid Intelligence. Performance is indicated as proportion of correct responses, except for HP_res and SA_res where residuals were used as difference scores (see Methods for more details). For the composite task, averages for differences score (HP-diff = CC-IC) are also provided since this index provides a more intuitive measure of the strength of holistic processing.

*Because of missing values, *N* = 98 for acuity (See Participants for more details)

### Multiple regression models

The predictions outlined in the Introduction were tested using two series of regression models (see Table 2). Spearman’s rank correlations are provided in Additional file 1: Tables 1, 2. Model 1 (M1) examines the extent to which each task measures face processing independent of general cognitive abilities. Model 2 (M2) examines relationships between face processing tasks. Given that correlations between acuity and the CFMT/GFMT were not significant (see Additional file 1: Table 1, 2), we did not include visual acuity in Model 1 to facilitate comparison with YA.

**Table 2:** Multiple regression models

CFMT								
		*b*	*SE*(b)	*t*(136)	*p*	*R*	*Corr.R*^2^	*p*
*OA*								
M1	SA	− 0.12	0.09	− 1.21	0.22			
	MR	0.00	0.10	− 0.05	0.95			
	FI	0.20	0.10	1.87	0.06	.06	.03	.09
M2	GFMT^***^	0.33	0.09	3.43	< .001			
	HP	− 0.06	0.09	− 0.65	0.51	.10	.08	< .01
*YA*								
M1	SA	0.05	0.08	0.67	.50			
	MR^**^	0.27	0.08	3.07	< .01			
	FI^*^	− 0.17	0.08	− 2.03	< .05	.07	.05	< .05
M2	GFMT^***^	0.40	0.07	4.96	< .001			
	HP	0.08	0.13	1.03	.30	.18	.17	< .001

GFMT								
		*b*	*SE*(b)	*t*(97)	*p*	*R*	*Corr.R*^2^	*p*
*OA*								
M1	SA	− 0.00	0.09	− .007	0.99			
	MR	0.14	0.09	1.49	0.13			
	FI^***^	0.36	0.10	3.67	< .001	.20	.17	< .001
M2	CFMT^***^	0.31	0.09	3.43	< .001			
	HP^*^	0.21	0.09	2.35	< .05	.15	.13	< .001
*YA*								
M1	SA	− 0.02	0.08	− 0.23	.81			
	MR^*^	0.22	0.08	2.55	< .05			
	FI	− 0.01	0.08	− 0.18	.85	.04	.02	.07
M2	CFMT^***^	0.39	0.07	5.10	< .001			
	HP^*^	0.15	0.07	2.00	< .05	.20	.19	< .001

The table shows standardized *(b)* coefficients with their standard errors, *t—*statistic with significance level, multiple correlation coefficient, and determination coefficient. ΔCorr.R^2^ is the change in corr. R^2^ when a further covariate enters the model. *M1* Model 1, *M2* Model 2, *CFMT* Cambridge Face Memory Test, *GFTM* Glasgow Face Matching Test, *HP* Holistic Processing, *SA* Selective Attention, *MR* Mental Rotation, *FI* Fluid Intelligence

For OA, general cognitive abilities significantly predicted performance on the GFMT, with fluid intelligence as a significant predictor of performance (M1: 17% of variance accounted for by the model). For the CFMT, the model did not reach significance. Models testing associations between face tasks (M2) were significant for both the CFMT (8% of variance accounted for) and GFMT (13% of variance accounted for).

For YA, general cognitive abilities significantly predicted performance on the CFMT, with mental rotation and fluid intelligence as significant predictors. However, the model only accounted for 5% of variance in the data. For the GFMT, the model was almost statistically significant (with mental rotation as a significant predictor), but the model only accounted for 2% of variance. Finally, models testing associations between face tasks (M2) were significant for both the CFMT (17% of variance accounted for) and GFMT (17% of variance accounted for).^4^

## Discussion

### Age-related differences in face-identity processing

Correlations and group differences reveal significant age-related decline for both the CFMT and GFMT. Hence, aging has a negative impact on both perceptual (matching) and memory components of face-identity processing. These findings are consistent with other studies showing significant cross-sectional group differences in performance on a variety of face matching and face memory tasks as early as 50 years of age (e.g., Boutet et al., 2015; Bowles et al., 2009; Verhallen et al., 2017) and a peak in performance at about 30 years of age (Germine et al., 2011; Susilo et al., 2013). As reported elsewhere (e.g., Boutet & Meinhardt-Injac, 2018; Hildebrandt et al., 2011), holistic processing was similar in YA and OA, suggesting that changes in this ability are not the source of age-related impairments in face processing. Associations between holistic processing, the CFMT, and GFMT are further discussed below.

### Associations between tests of face-identity processing and general cognitive abilities

In OA, performance on measures of general cognitive abilities did not predict performance on the CFMT. Fluid intelligence was a significant predictor of performance on the GFMT with the model accounted for 17% of variance. In YA, performance on measures of general cognition significantly predicted performance on the CFMT, albeit the model only accounted for a small portion of variance (5%). Reliability of the CFMT was good in both age groups. Reliability of the GFMT was questionable in OA and acceptable in YA.

As far as we know, our study presents the first evidence of the specificity of the CFMT in a large sample of OA. Similar results have been reported for YA (Bowles et al., 2009; Verhallen et al., 2017). Hence, it appears that the CFMT is a suitable test for measuring face memory in OA. One important issue is whether existing CFMT cut-off scores for the diagnosis of prosopagnosia are appropriate in older adults. Using two standard deviations from the mean as a cut-off, 1.7% of older adults would be diagnosed with prosopagnosia in our sample. This prevalence rate is comparable to prevalence of about 2% reported in YA (e.g., Grüter et al., 2008). However, using YA values to determine a cut-off score would have led to a diagnosis of prosopagnosia for 16% of the OA sample. Bowles et al. (2009) reported a similar finding. Our study therefore confirms the importance of using age-appropriate data when determining cut-off scores for the CFMT. Assuming that our results for the CFMT are replicated, it will be important for future research to collect data from a very large sample of individuals aged 50 + to establish norms for this population (e.g., see Wilmer et al., 2013 for a similar approach with younger adults).

With regards to the GFMT, results of past research are inconsistent. Verhallen et al. (2017) and Bowles et al. (2009) did not find significant relations between the GFMT and measures of general cognitive function. McCaffery et al. (2018) reported significant correlations between the GFMT and executive functions. It is important to note that all of these focused on YA. We found relations between the GFMT and fluid intelligence in OA. Hence, there appears to be some overlap between face matching as measured by the GFMT and general cognitive abilities in both YA and OA. These results may arise from poor test construction and/or from the specificity of underlying cognitive mechanisms. With regards to test construction, internal consistency was poorer for the GFMT, which suggests that some of this co-variability might be related to the psychometric properties of the test.

With regards to underlying cognitive mechanisms, putative determinants of performance on face processing tests include fluid intelligence (Gignac et al., 2016), mental rotation (our findings), general matching and recognition abilities (McCaffery et al., 2018), and speed of processing (Hildebrandt et al., 2011; Schretlen et al., 2001). Mental rotation ability was a significant predictor of performance on both tasks in YA, albeit a small amount of variance was accounted for by the model for the CFMT. One might speculate that older adults have less of a tendency to rely on mental rotation to perform face tasks because this ability declines with age (Techentin et al., 2014). Fluid intelligence was a significant predictor of performance for the GFMT in OA. Associations between the CFMT and fluid intelligence have also been reported in YA (Gignac et al., 2016). As a whole, this research suggests that some aspects of face-identity processing measurement are associated with general cognitive abilities.

Basic visual abilities did not significantly correlate with performance on the CFMT/GFMT. Past research has produced inconsistent results with some studies showing associations between face-identity processing and vision (e.g., Boutet et al., 2020; Owsley et al., 1981) but not others (Cronin-Golomb et al., 2007). Differences in study design and visual measures utilized across studies probably account for these differences. Most notable are differences between experimental vs. correlational designs (e.g., Cronin-Golomb et al., 2007 vs. Boutet et al., 2020) as well as measures of visual abilities. For example, contrast sensitivity thresholds vary depending on lighting conditions (e.g., Bühren et al., 2006), which may affect the validity of measures taken in the laboratory and subsequent correlations with measures of face processing.

### Associations between measures of face-identity processing

For both age groups, models testing associations between measures of face processing were statistically significant. These models also consistently accounted for more variability in the data than models linking each face task with general cognitive abilities. This pattern of results lends support for the existence of a general face factor *f* (McCaffery et al., 2018; Verhallen et al., 2017; Wilmer et al., 2014). Including OA in our sample allows us to extend this conclusion across the adult lifespan.

With regards to holistic processing, for both age groups performance on the composite task was a significant predictor of performance on the GFMT, but not the CFMT. Konar et al. (2013) reported that performance on a comparable face matching task was significantly correlated with HP in OA but not YA. In YA, studies that have examined the relationship between face recognition and HP have produced mixed results (DeGutis et al., 2013; Nelson et al., 2016; Rezlescu et al., 2017; Richler et al., 2011; Verhallen et al., 2017). Inconsistencies in the literature may stem, in part, from the use of different measures of holistic processing (see, e.g., Rossion, 2013; Richler & Gauthier, 2014, for opposite views on of the best way to measure the composite effect). To further complicate matters, HP tends to be larger in OA than YA (present study; Boutet & Meinhardt-Injac, 2018; Boutet et al., 2015). The phenomenon of impaired face processing alongside enhanced HP suggests that HP is necessary but not sufficient for successful face recognition (e.g., Boutet et al., 2020; Watson, 2013). While additional research is needed to better understand this phenomenon, it may help explain why relationships between face recognition and HP vary across studies.

### Limitations

It may be argued that constructs such as object perception or processing speed should have been included in our measures of general cognition (e.g., Gauthier, 2018; Hildebrandt et al., 2011; Schretlen et al., 2001). Our choice of measures of general cognition was motivated by practical and theoretical considerations. In individual differences studies, a large number of participants needs to be tested each time a new task is added in order to retain power. This sets a limit on the number of tasks that can be included in any study, especially when testing OA. We selected measures that have been linked to aging and/or face processing in past research and therefore that were likely to be important predictors of performance in OA.

We utilized the CFMT and GFMT in their original formats, which entailed presenting young faces and unfamiliar faces. Future research should aim to adapt tests of face processing to older populations by, for example, including faces of OA. This would enhance ecological validity and avoid potential biases in performance (i.e., own-age bias, see reviews by Rhodes & Anastasi, 2012; Schaich et al., 2016).

### Conclusion

Our results extend the notion of a general face processing factor (McCaffery et al., 2018; Verhallen et al., 2017) across the adult lifespan. Our results also suggest that face memory abilities can be measured independent of general cognitive function in OA using the CFMT. Mixed-results have been reported for face matching abilities measured using GFMT: some studies have reported some degree of specificity (Bowles et al., 2009; Verhallen et al., 2017), while others show associations between the GFMT and general cognitive abilities (our study, McCaffery et al., 2018). These findings complicate interpretation of GFMT scores, especially in OA where data are sparse. Discrimination is a key component of face recognition (McCaffery et al., 2018) and is more relevant than memory in certain professional contexts (e.g., passport officers, Burton et al, 2010). Future research should therefore seek to clarify the relationship, or lack therefore, between face matching and other cognitive functions.

Our literature search on individual differences and measurement of face processing abilities revealed only a handful of studies that have included adults aged 50 + in their sample. This highlights the need to include participants from across the adult lifespan to better characterize developmental changes in face-identity processing. Such research is needed to corroborate our findings, to identify measures of face processing abilities that are suitable in geriatric populations, and to expand our understanding of the impact of aging on social cognition.

## Supplementary Information

**Additional file 1**. Table 1. Mean reaction time (RTs) in milliseconds for younger (YA) and older adults (OA). Table 2 Spearman's rank correlations for the entire sample, younger adults and older adults

## Data Availability

The datasets generated during and/or analyzed during the current study are available from https://osf.io/gsv9k/. This experiment was not pre-registered.

## Footnotes

1 Cognitive and biological mechanisms specialized for processing human faces can also be involved in processing other image categories in experts (e.g., Gauthier, 2018; Haxby et al., 2000). However, for most humans, faces are the only category of expertise and hence the only category to elicit these specialized mechanisms (McKone et al., 2006).

2 These measures were administered as part of a larger project on social cognition. Given the practical difficulties associated with testing older adults on multiple tasks for long periods of time, and that a large additional number of participants needs to be added for each task to maintain acceptable power in regression analyses, we focus here on a limited set of measures that had the potential to influence performance on face perception tasks and yet had received little attention in the past.

3 There was an overrepresentation of female participants in our YA group (YA: 74% female; OA: 53%). There is some evidence that females are better than males at discriminating facial identity (reviewed by Herlitz & Lovén, 2013, but see, e.g., Verhallen et al., 2017). Exploratory analyses with YA did not reveal significant gender effects in any of the measures tested. We therefore conclude that gender differences are unlikely to have played an important role in our findings.

4 These two models are somewhat redundant because they encompass the same three tasks. Both are included because they are informative regarding predictors of performance on each task separately, and because their presentation facilitates comparisons across each task in the table.
