# Supplementary material for: Measurement of individual differences in face-identity processing abilities in older adults
Source: Cogn Res Princ Implic. 2021 Jul 18;6:48. doi: 10.1186/s41235-021-00310-4 (PMC8286909; doi:10.1186/s41235-021-00310-4)
Supplement: Supplementary file 1 — Additional file 1. Table 1. Mean reaction time (RTs) in milliseconds for younger (YA) and older adults (OA). Table 2 Spearman's rank correlations for the entire sample, younger adults and older adults [file 41235_2021_310_MOESM1_ESM.docx]

**Supplementary Materials**

Table 1.

Mean reaction time (RTs) in milliseconds for younger (YA) and older adults (OA)

|  | YA | | | | OA | | | | | |
| --- | --- | --- | --- | --- | --- | --- | --- | --- | --- | --- |
|  | *M* | *SD* | *Min* | *Max* | | *M* | *SD* | *Min* | *Max* |  |
| CFMT | 3 442 | 2263 | 94 | 53 623 | | 5 220 | 4 681 | 571 | 112 913 |  |
| GFMT | 6 079 | 4 783 | 79 | 40 856 | | 10 734 | 10 563 | 16 | 142 073 |  |
| HP | 1 092 | 2 014 | 97 | 92 025 | | 1 670 | 3 684 | 102 | 158 980 |  |
| SA | 969 | 993 | 71 | 3 461 | | 1013 | 811 | 352 | 6 122 |  |

*Note:* CFMT = Cambridge Face Memory Test; GFTM = Glasgow Face Matching Test; HP = Holistic Processing; SA = Selective Attention.

Table 2

*Spearman's rank correlations for the entire sample, younger adults and older adults*

|  | Age | CFMT | GFMT | HP | SA | MR |
| --- | --- | --- | --- | --- | --- | --- |
| Age |  |  |  |  |  |  |
| CFMT | -.35^***^ |  |  |  |  |  |
| GFMT | -.23^***^ | .43^***^ |  |  |  |  |
| HP | -.23^***^ | .21^**^ | .27^***^ |  |  |  |
| SA | .37^***^ | -.22^**^ | -15^**.^ | -.03^n.s.^ |  |  |
| MR | -.32^***^ | .27^***^ | .28^***^ | .17^**^ | -.14^*^ |  |
| FI | -.68^***^ | .33^***^ | .30^***^ | .29^***^ | -.37^***^ | .49^***^ |

*Younger adults group only*

|  | CFMT | GFMT | HP | SA | MR |
| --- | --- | --- | --- | --- | --- |
| CFMT |  |  |  |  |  |
| GFMT | .41^***^ |  |  |  |  |
| HP | .21^*^ | .22^**^ |  |  |  |
| SA | .04^n.s.^ | .04^n.s.^ | .09^n.s.^ |  |  |
| MR | .21^**^ | .19^*^ | .13^n.s.^ | .07^n.s.^ |  |
| FI | -.07^n.s.^ | .07^n.s.^ | .16^n.s.^ | .04^n.s.^ | .38^***^ |

*Older adults group only*

|  | CFMT | GFMT | HP | SA | MR | Visual Acuity |
| --- | --- | --- | --- | --- | --- | --- |
| CFMT |  |  |  |  |  | .09 |
| GFMT | .32^***^ |  |  |  |  | .07 |
| HP | .02^n.s.^ | .23^*^ |  |  |  | .17 |
| SA | -.20^*.^ | -.14^n.s.^ | .02^n.s.^ |  |  | -.18 |
| MR | .04^n.s.^ | .25^*^ | .10^n.s.^ | -.13^n.s.^ |  | .05 |
| FI | .30^**^ | .42^***^ | .15^n.s.^ | -.15^n.s.^ | .39^***^ | .04 |

*Note.* *** *p* ≤ .001, ** *p* ≤ .01, * *p* ≤ .05, ^n.s.^ *p* > .05. (CFMT = Cambridge Face Memory Test; GFTM = Glasgow Face Matching Test; HP = Holistic Processing; SA = Selective Attention; MR = Mental Rotation; FI = Fluid Intelligence). Spearman’s correlations were computed because the data violated the assumption of normality. Note that correlation between visual acuity and contrast sensitivity are reported for a sample of older adults *N* = 97 because of missing values, as compared to full sample of *N*=102 participants in other cells.
